# Challenges in diagnostic accuracy studies in primary care: the fecal calprotectin example

**DOI:** 10.1186/1471-2296-14-179

**Published:** 2013-11-25

**Authors:** Gea A Holtman, Yvonne Lisman-van Leeuwen, Boudewijn J Kollen, Johanna C Escher, Angelika Kindermann, Patrick F van Rheenen, Marjolein Y Berger

**Affiliations:** 1 Department of General Practice, FA21, University of Groningen, University Medical Center Groningen, PO Box 196, 9700 AD Groningen, The Netherlands; 2 Department of Pediatric Gastroenterology, Erasmus MC-Sophia Children’s Hospital, PO Box 2060, 3000 CB Rotterdam, The Netherlands; 3 Department of Pediatric Gastroenterology, Emma Children’s Hospital/Academic Medical Center, PO Box 22700, 1100 DE Amsterdam, The Netherlands; 4 Department of Pediatric Gastroenterology, Beatrix Children’s Hospital/University of Groningen, University Medical Center Groningen, PO Box 30001, 9700 RB Groningen, The Netherlands

**Keywords:** Primary care, Risk of bias, diagnostic research, Calprotectin, Inflammatory bowel disease

## Abstract

**Background:**

Low disease prevalence and lack of uniform reference standards in primary care induce methodological challenges for investigating the diagnostic accuracy of a test. We present a study design that copes with these methodological challenges and discuss the methodological implications of our choices, using a quality assessment tool for diagnostic accuracy studies (QUADAS-2).

**Design:**

The study investigates the diagnostic value of fecal calprotectin for detecting inflammatory bowel disease in children presenting with chronic gastrointestinal symptoms in primary care. It is a prospective cohort study including two cohorts of children: one cohort will be recruited in primary care and the other in secondary/tertiary care. Test results of fecal calprotectin will be compared to one of the two reference standards for inflammatory bowel disease: endoscopy with histopathological examination of mucosal biopsies or assessment of clinical symptoms at 1-year follow-up.

**Discussion:**

According to QUADAS-2 the use of two reference standards and the recruitment of patients in two populations may cause differential verification bias and spectrum bias, respectively. The clinical relevance of this potential bias and methods to adjust for this are presented. This study illustrates the importance of awareness of the different kinds of bias that result from choices in the design phase of a diagnostic study in a low prevalence setting. This approach is exemplary for other diagnostic research in primary care.

## Background

In primary care, patients often present with non-specific symptoms and the incidence of severe illnesses is low. Differentiating between innocent symptoms and a rare, but serious organic disease is a diagnostic dilemma for the primary care physician (PCP). Unnecessary referrals and diagnostic testing need to be balanced against the risk of missing a diagnosis and introduction of an unacceptable long diagnostic delay. In primary care, both the PCP and the patient would greatly benefit from simple, non-invasive and specific screening tests. However, many of these tests are not validated in primary care.

An example of such a diagnostic dilemma are children presenting with chronic or recurrent gastrointestinal symptoms. This clinical picture is common, but few children will actually have inflammatory bowel disease (IBD), which includes Crohn’s disease and ulcerative colitis. The incidence of non-specific abdominal pain in Dutch children is 2500/100,000 per year, while the incidence of IBD is 5.2/100,000 per year [1,2]. Clinical symptoms in children with IBD are often non-specific and show substantial overlap with functional gastrointestinal disorders [3]. In European secondary and tertiary care facilities the measurement of calprotectin in stool is used as an effective triage method for endoscopy, which is the reference standard for the diagnosis of IBD [4]. Calprotectin is a marker of inflammation that can be measured by using a simple non-invasive test [5], but has never been evaluated in children in a primary care setting [6-8]. The different patient spectrum in primary care has consequences for the pre-test probability and test characteristics. Before calprotectin testing can be recommended to distinguish functional from organic gastrointestinal disorders at the primary care level, information is required on the predictive value of fecal calprotectin at the primary care level.

The preferred design to evaluate the diagnostic value of fecal calprotectin in children with chronic gastrointestinal symptoms would be a cross-sectional study. Such a design has two methodological challenges. Firstly, the design of a diagnostic study for rare diseases requires a large population in order to identify a sufficient number of children with IBD; the financial and logistic exercise involved makes such a study infeasible [9]. Secondly, the preferred reference standard to detect IBD is endoscopy [10]; but it is unethical to perform this invasive test in children with a low likelihood of organic gastrointestinal disease.

Here we present an example of a design that copes with these methodological challenges. The methodological implications of applied design choices are examined using an evidence-based quality assessment tool for diagnostic accuracy studies (QUADAS-2) [11].

## Design

### Design and setting

**Figure 1:**
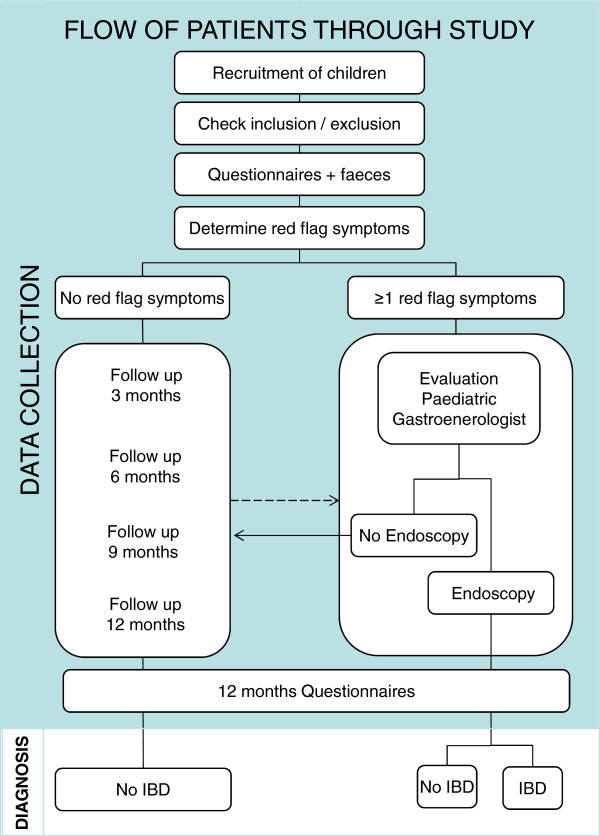
**Flow chart of the DOK study.** The PCP or pediatric gastroenterologist selects eligible children. At baseline inclusion, exclusion criteria and red flag symptoms are determined. The parents and child ≥10 years complete two questionnaires, i.e. a Questionnaire on Pediatric Gastrointestinal Symptoms (QPGS) and a symptoms questionnaire, in addition feces (parasites and colon pathogens) are obtained. Children meeting 1 ≥ red flag symptoms are evaluated for eligibility for endoscopy by a pediatric gastroenterologist. Children without red flag symptoms receive a 1-year follow-up. The arrows indicate that the PCP can refer a child during follow-up for endoscopic evaluation and the children who are not eligible for endoscopy receive a follow-up. After 1 year, information about diagnosis and clinical symptoms is collected based on the two above-mentioned questionnaires.

The DOK (*Darm Onderzoek bij Kinderen*: Bowel Research in Children) study is a prospective cohort study with a follow-up period of one year, also known as a delayed type cross-sectional study [12]. The study consists of two prospective cohorts. We will recruit a primary care cohort of children presenting consecutively in primary care in the northern part of the Netherlands (PCP cohort). A second cohort consists of children that will be referred to secondary and tertiary care facilities across the Netherlands (Hospital cohort). The index test is fecal calprotectin and the two reference standards for IBD are endoscopy with histopathological examination of mucosal biopsies, or (in children without indication for endoscopy) assessment of clinical symptoms at 1-year follow-up (Figure 1) [4,13]. The DOK study was approved by the Medical Ethics Review Committee of the University Medical Center Groningen. Written informed consent will be obtained from the parents and from the child if aged ≥12 years. Inclusion started in June 2011.

### Study population

Children aged 4-18 years presenting with chronic diarrhea (≥2 weeks diarrhea or ≥2 episodes of diarrhea in the past 6 months) or recurrent abdominal pain (≥2 episodes of abdominal pain in the past 6 months) will be eligible for participation. Diarrhea was defined as moderately to watery loose stools matching score 5, 6 or 7 of the Bristol Stool Form Scale [14]. One episode is defined as 3 days or more.

Exclusion criteria are: a previously established diagnosis of chronic organic gastrointestinal disease; a complete evaluation in the past 6 months for abdominal symptoms including endoscopy; chronic use of antibiotics, non-steroidal anti-inflammatory drugs (NSAIDs) or oral corticosteroids (defined as daily use during ≥3 months/year); fecal calprotectin test in the past 6 months, and difficulty in understanding questionnaires. The number of patients not participating due to the exclusion criteria or refusal are anonymously recorded, including the patient characteristics and, if available, the reason for non-participation.

### Measurements

#### Physical examination

The PCP or pediatric gastroenterologist performs a structured physical examination and assesses extra-intestinal symptoms and peri-anal lesions according to the Dutch diagnostic guideline [15]. The participating PCPs receive training on structured physical examination of children with symptoms suggestive of IBD.

#### Questionnaire on pediatric gastrointestinal symptoms

The Dutch version of the Questionnaire on Pediatric Gastrointestinal Symptoms ROME III (QPGS-RIII) [16] is completed, by the patient or a parent at baseline and at 12 months follow-up. The QPGS-RIII consists of two reports, a parent report for children aged 4-18 years and a self-report for children aged ≥10 years. The questionnaire has been translated into Dutch. The English version of QPGS has good content validity and test-retest reliability [17,18].

#### Blood and fecal tests

In the blood sample hemoglobin, erythrocyte sedimentation rate, C-reactive protein, platelet count and serology tests for celiac disease (IgA tissue transglutaminase antibodies) are measured. Feces is tested for colon pathogens (*Salmonella enterica*, *Campylobacter jejuni*, *Shigella spp/EIEC*, STEC) and parasites (*Giardia lamblia, Cryptosporidium spp*, *Dientamoeba fragilis*, *Entamoebe histolytica*) with the real-time multiplex PCRs [19]. Blood and feces tests are performed at local certified laboratories. If a child is using NSAIDs, antibiotics or oral corticosteroids for short-term use (<3 months), the collection and testing of feces is postponed until the end of that treatment.

#### Fecal calprotectin

After baseline assessments the patients send the feces sample by pre-stamped return envelope to the laboratory where the samples are stored at -80°C. At the end of the data collection period the samples are defrosted before analysis. Fecal calprotectin is measured by means of a commercially available quantitative enzyme-linked immunosorbent assay (ELISA) [20,21]. In accordance with the manufacturer’s guidelines, values above 50 μg/g feces are regarded as positive.

#### Red flag symptoms

In all children red flag symptoms of IBD will be searched for using a structured evaluation form (Table 1). Children who fulfill the inclusion criteria and have ≥1 red flag symptoms are referred to a pediatric gastroenterologist who will decide whether the child requires endoscopic examination [15]. This decision will be based on the medical history, physical examination and blood testing. Children without red flag symptoms, or those who are not eligible for endoscopy will be followed for one year.

**Table 1:** Red flag symptoms of IBD

**Red flag symptom**	**Measurement**	**Positive**
Growth failure	Growth calculator	Height for age < -1 SDS
Involuntary weight loss	History	Involuntary decrease in weight
Rectal blood loss	History	Rectal blood loss with defecation
Positive family history of inflammatory bowel disease	History	First-degree relatives
Extra-intestinal symptoms	Physical examination	Eyes (episcleritis, scleritis, uveitis), skin (erythema nodosum, pyoderma gangrenosum, psoriasis), mouth ulcers, finger clubbing, arthritis
Peri-anal lesions	Physical examination	Skin tags, hemorrhoids, fissures, fistulas, abscess
Anemia (Hb)	Hematology	4-12 years < 7.1 mmol/l,
		boy 12-18 years <8.1 mmol/l,
		girl 12-18 years <7.4 mmol/l [22]
CRP	Chemistry	> 10 mg/l [23]
ESR	Hematology	≥ 20 mm/h [23,24]
Platelets	Hematology	> 450 x 10^9^ /l [24]

SDS = standard deviation score; Hb = hemoglobin; CRP = C-reactive protein; ESR = erythrocyte sedimentation rate.

#### Endoscopy

Endoscopy is performed under full anaesthesia or deep sedation by an experienced pediatric gastroenterologist and entails oesophagogastroduodenoscopy and ileocolonoscopy. Two biopsies of each intestinal segment are taken. The histopathological examination will be performed by an experienced gastrointestinal histopathologist. IBD is classified according to the Paris classification [10].

#### Follow-up

Follow-up is done using a symptom questionnaire that was developed for the study in cooperation with pediatric gastroenterologists and PCPs. This questionnaire will be completed by the parent or child (if aged ≥10 years) at 3, 6, 9 and 12 months follow-up. The PCP will perform a structured physical examination to assess red flag symptoms in children with clinical symptoms at 12 months. Those with ≥1 red flag symptoms at 12 months will be referred to a pediatric gastroenterologist to determine a diagnosis.

#### Blinding

The pediatric gastroenterologists, pathologists, PCPs and researchers will be blinded to the outcome of the fecal calprotectin test. The laboratory technician will be blinded for the clinical characteristics of the child and the result of endoscopy.

### Outcome

IBD is confirmed when the endoscopic picture and the histopathological picture match. Absence of IBD is defined as a negative endoscopic and histopathological examination, or when there was no indication to perform endoscopy at all during the 12 months follow-up. Besides, all children without red flag symptoms at 12 months follow-up are considered not to have IBD [13].

### Sample size

Based on available literature we expect to find a specificity of 93% in the PCP cohort [7,25-27]. To estimate the specificity and a 95% confidence interval (CI) spanning 5%, we assume a maximum IBD incidence of 5 per 100 children with gastrointestinal complaints and a loss to follow-up of 10%, we will then need a sample size of 118 children in the PCP cohort. In a worst case scenario with a specificity of 75%, a sample size of 118 children will widen the 95% CI to 8% [28].

Sensitivity will be calculated in children with red flag symptoms (PCP and Hospital cohort). Based on an expected sensitivity of 95% we need to include 73 children with IBD in order to estimate the sensitivity and a 95% CI spanning 5% [7,26,27]. With a prevalence of 80% IBD and a loss to follow-up of 10% we need to include 100 children with red flag symptoms. The prevalence of IBD is difficult to estimate; with a prevalence of 20% the spanning of the 95% CI of the sensitivity will widen to 10% [28].

### Statistical analyses

Specificity of fecal calprotectin for IBD in primary care will be calculated by dividing the number of negative fecal calprotectin tests by the total number of children without IBD included in the PCP cohort. Sensitivity will be calculated by dividing the number of positive fecal calprotectin tests by the total number of children with IBD in children with red flag symptoms of both the PCP and Hospital cohort. The estimates of specificity and sensitivity will be reported as percentages with 95% CIs.

## Discussion

### Assessing the risk of bias

To address the risk of bias in our study design and the applicability of the results we applied the QUADAS-2 checklist [11] that includes four domains: patient selection, index test, reference standard and flow and timing (flow of patients through the study and timing of the index test and reference standard). Each domain was scored as low or high risk of bias, based on the answers to the signaling questions. If all answers concerning a domain are “yes”, the risk of bias can be judged as low. If any signaling question is answered “no” the risk of bias can be judged as high. The first two domains were scored as low or high concerns regarding applicability. Two items were excluded because one item assessed heterogeneity between studies, which is only applicable in systematic reviews. The second item asked whether all patients are included in the analysis, which can only be assessed after completion of the study. The results of the QUADAS-2 assessment are shown in Table 2.

**Table 2:** Quality assessment of the DOK study design (QUADAS-2)

**Signaling questions**	**Answer**	**Risk of bias/applicability**	**Planned adjustment**
**Domain 1: Patient selection**			
**Risk of bias**		**Low risk**	
Is a consecutive sample of patients enrolled?	Yes		
Is a case-control design avoided?	Yes		
Does the study avoid inappropriate exclusions?	Yes		
**Applicability**		**High concern**	- Magnitude will be evaluated
Are there concerns that the included patients and setting do not match the topic of our study (patients had symptoms suggestive of inflammatory bowel disease in primary care)?			
**Domain 2: Index test**			
**Risk of bias**		**Low Risk**	
Are the index test results interpreted without knowledge of the results of the reference standard?	Yes		
If a threshold was used, is it pre-specified?	Yes		
**Applicability**		**Low Concern**	
Are there concerns that the index test, its conduct, or interpretation differ from the topic of our study (fecal calprotectin was measured with ELISA)?			
**Domain 3: Reference standard**			
**Risk of bias**		**High risk**	
Is the reference standard likely to correctly classify the target condition?	No		- Probably not clinically relevant
			- Adjustment in analysis [30,31]
Are the reference standard results interpreted without knowledge of the results of the index test?	Yes		
**Domain 4: Flow and timing**			
**Risk of bias**		**High risk**	
Is there an appropriate interval between index test and reference standard?	No		- Represents care as usual
			- Repeated measurement index test before endoscopy
Do all patients receive a reference standard?	Yes		
Do all patients receive the same reference standard?	No		- Adjustment in analysis [30,31]

### Risk of bias

#### Problems with the reference standard

A perfect reference standard in a diagnostic accuracy study is said to fulfill three criteria: “1) The reference standard provides error-free classification of all subjects. 2) The same reference standard is used to verify all index results. 3) The index test and reference standard can be performed within a short interval to avoid changes in target condition status [29].”

Risk of bias in the DOK study is related to the choice of the reference test, which is not the same for all included patients (differential verification bias) [30]. In addition, follow-up is not considered a reference standard for IBD in daily practice. This choice may lead to missed diagnoses and will influence the estimates of sensitivity and specificity. We chose a differential verification design, because it is unethical to perform endoscopy in children with a low likelihood of organic gastrointestinal disease. Therefore, children who have a low IBD risk receive a follow-up of one year, which is considered to be a suitable period [13]. On the opposite side, it might be possible that even more children will be identified because, using a 1-year follow-up, children with initially mild IBD can be detected when they have an aggravation of symptoms later in time. These children could have been missed when endoscopy was performed at initial presentation. Children in whom endoscopy was not indicated during the 1-year follow-up (either because they no longer have symptoms or because their red flag symptoms are not suggestive for IBD) are considered not to have IBD. The probability that we will miss a child with IBD is considered to be extremely low [13]. Adjustment for differential verification bias will be made, if possible, using a Bayesian approach [30,31].

The patient flow of the DOK study could introduce bias. A delay of one month between stool sample collection and reference standard is considered to be an appropriate time period. In children of the Hospital cohort the interval between fecal sampling and endoscopy will generally be less than one month. For referred children in the PCP cohort this interval is likely to exceed the period of one month. To investigate whether the concentration of fecal calprotectin accurately measures the same outcome as endoscopy, feces will be collected again shortly before endoscopy. In children not referred to a secondary or tertiary care facility the period between fecal sampling and reference test will be one year. During this period the calprotectin concentration may change and, therefore, the initial test result will no longer be related to the outcome of endoscopy. This will underestimate sensitivity and specificity. Here we adopt a pragmatic approach. We want to establish whether fecal calprotectin can serve as a screening test in children who are presenting for the first time to their PCP. A negative fecal calprotectin value at the start of the study, and a positive endoscopic result at the end of the study, should be considered as a false-negative test result.

### Applicability of study results

#### Problems with the patient selection

Test characteristics should be evaluated in a clinically relevant population [32]. In the DOK study the patients with symptoms suggestive of IBD will be recruited in both primary and secondary/tertiary care. Spectrum bias is to be expected as our patient cohorts will have different characteristics [32]. To reduce the risk of spectrum bias one should ideally only include children who initially presented at the primary care level. The low prior probability in this setting makes such a study design infeasible with considerable financial and logistic problems [9]. We decided to use a pragmatic design, based on the following assumptions: in case of a very low prior probability of IBD, a PCP wants to avoid unnecessary referrals. The false-positive rate thus needs to be low. Therefore, we will evaluate specificity of fecal calprotectin in children presenting in primary care. In children with red flag symptoms, a PCP wants to rule out IBD and minimize false-negative results. Sensitivity will thus be evaluated in children referred to secondary or tertiary care (children with red flag symptoms in PCP cohort and Hospital cohort).

We assume that this sensitivity is a representative estimate for sensitivity measured in children with red flag symptoms in primary care. This implies two additional assumptions: 1) in both cohorts the ratio IBD/non-IBD in children with red flag symptoms will be comparable (which we will test); 2) children with red flag symptoms of both cohorts are comparable (which we will test by comparing the clinical characteristics). In case the children from the Hospital cohort are more severely ill, sensitivity will be overestimated. Heterogeneity can then be assessed by subgroup analyses of the test performance.

## Conclusion

Low disease prevalence and lack of uniformity in reference standard in primary care creates methodological challenges in primary care level diagnostic accuracy studies. We presented a pragmatic design in which the magnitude of potential bias will be assessed and controlled. Awareness of the potential biases and its implications allows to discuss possible solutions and to overcome such bias. The validity of diagnostic research at the primary care level may be considerably improved with the proposed design.

## Abbreviations

PCP: Primary care physicians; IBD: Inflammatory bowel disease; QUADAS: Quality assessment tool for diagnostic accuracy studies; DOK: *Darm Onderzoek bij Kinderen*: Bowel Research in Children; NSAIDs: Non-steroidal anti-inflammatory drugs; QPGS-RIII: Questionnaire on Pediatric Gastrointestinal Symptoms ROME III; ELISA: Enzyme-linked immunosorbent assay; CI: Confidence interval.

## Competing interests

The authors declare that they have no competing interests.

## Authors’ contributions

MYB, YLvL and GAH were responsible for study design, and conceptualization, as well for the interpretation of data, drafting and revising of the manuscript. PFvR, JCE, AK were responsible for conception, design and revising the manuscript critically for important intellectual content. BK was responsible for the interpretation of data and revising of the manuscript. All authors have read and approved the final manuscript.

## Pre-publication history

The pre-publication history for this paper can be accessed here:

http://www.biomedcentral.com/1471-2296/14/179/prepub
